# Potato leaves disease classification based on generalized Jones polynomials image features

**DOI:** 10.1016/j.mex.2025.103421

**Published:** 2025-06-06

**Authors:** Ala’a R. Al-Shamasneh

**Affiliations:** Department of Computer Science, College of Computer & Information Sciences, Prince Sultan University, Rafha Street, Riyadh 11586, Saudi Arabia

**Keywords:** Potato disease detection, Feature extraction, Generalized jones polynomials, Classification, plant images, Classification of potato diseases from images

## Abstract

The detection of plant diseases in the modern era offers a promising first step toward sustainable agriculture and food security. Plant physiology can be studied quantitatively thanks to advances in imaging and computer vision. Conversely, manual interpretation requires a great deal of labor, knowledge of plant diseases. Numerous innovative methods for identifying and classifying particular diseases have been widely used. In order to diagnose potato diseases more accurately and quickly using a machine learning model, this study uses a new feature extraction method based on GJPs image features. The methodology of this study relies on:

• Modules for preprocessing, feature extraction, dimension reduction, and classification.
• Generalized jones polynomials as new image features method is used to extract the texture features from potato images for diagnosing potato diseases.

The data used in this model were collected from the plant village image dataset using samples of potato leaves. Using an SVM classifier on potato leaf images, the disease was accurately identified in 98.45 % of cases. The recommended feature extraction technique can reduce financial loss while also assisting in the efficient management of plant diseases, enhancing crop productivity and ensuring food security.

## Graphical abstract

**Figure:**
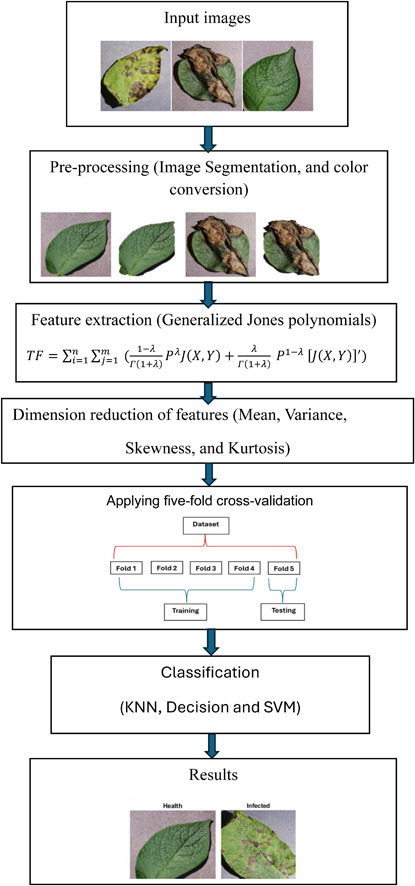

Specifications table

**Table:** 

Subject area:	*Computer Science*
More specific subject area:	Image processing
Name of your method:	Classification of potato diseases from images
Name and reference of original method:	Not applicable
Resource availability:	Plant Village image dataset

## Background

With the expansion of the agriculture sector, increasing agricultural productivity can both directly strengthen the economy and indirectly reduce poverty. Numerous illnesses that have affected crop production have resulted in significant losses in the production of both fresh and processed products.

One of the four major food crops in the world, potatoes rank among the top ten nutrient-dense, healthful foods and are a high-yield crop with future growth potential. It has been highly valued globally because of its long industrial chain, full nutritional content, wide adaptability, high yield, and stability.

The leaves are the most vulnerable portion of a plant and show symptoms of disease initially. It is possible to identify a plant disease by looking for certain signs. A closer look reveals the underlying reason, even if dark patches or yellowing on leaves are early signs of many illnesses. Crops require routine inspections throughout their growth cycle in order to identify any possible illnesses or diseases. Prior to harvesting, this procedure is required to make sure the crops are healthy and free of any dangerous substances [1].

The two most damaging foliage diseases to potato crops are early and late blight. Potatoes are susceptible to both early and late blight at any point in their growth cycle. The early stages of late blight are patches of light green that quickly turn brownish black. Similarly, early blight has irregular or round patches that are dark brown or black in color. It is crucial to recognize and detect potato leaf diseases in order to effectively and promptly prevent and control disease [2]. Saudi Arabia is currently experiencing a high demand for agriculture as a result of shifting consumer habits and rising consumer awareness. One of the principal crops grown in Saudi Arabia is the potato [3].

Farmers use manual methods to identify crop illnesses, which not only takes up valuable time but also introduces subjectivity and the possibility of errors because of human participation [4].

Utilizing computer intelligence and image processing techniques, which may profit from precise and effective crop disease diagnosis, is now a viable way to handle this problem [5,6].

Several Artificial Intelligence (AI) methods have been applied in precision agriculture to automatically detect and identify potato leaf disease [7]. These methods are inspired by the remarkable achievement of AI technologies, including the traditional Machine Learning (ML) algorithms and the latest Deep Learning (DL) techniques [8,9]. Machine vision technologies and DL algorithms are currently widely used for plant disease classification tasks [10].

Several approaches for potato leaf disease classification have been proposed based on either image processing and machine learning or deep learning to construct automated disease detection systems [2]. Based on the body of existing research, this study proposed a new feature extraction technique utilizing Genderized Jones Polynomials (GJPs) for the detection of potato diseases in images.

Conventional techniques for diagnosing potato diseases depend on the knowledge of plant specialists and farmers. Potato diseases can have severe effects on yield because inexperienced farmers frequently misidentify the types and stages of the disease Image processing technology advancements have made it possible for technicians and scientists to extract image attributes from potato leaves in order to identify potato illnesses. Thanks to developments in image processing technology, scientists and technicians can now identify potato diseases by extracting image features from potato leaves [11]. By helping farmers and agricultural specialists identify and treat diseases early on, this technology may increase crop yield and lower losses.

Applications based on image processing for the detection and categorization of plant diseases are currently the subject of extensive research. Many researchers have looked at various imaging modalities and approaches to obtaining disease characteristics [10,12].

In order to enable the diagnosis of potato diseases from leaf images, Monzurul Islam et al. [13] presented an approach that combines image processing and machine learning. The Gray-Level Co-occurrence Matrix (GLCM) was used to extract statistical texture features like contrast, correlation, energy, and homogeneity. In addition, the color plane histograms were used to compute numerical indicators such as energy, skew, entropy, mean, and standard deviation. With a 95 % accuracy rate, the SVM classifier is able to classify diseases across 300 images.

Using a publicly accessible plant village database and classifier algorithms to distinguish between diseased and healthy leaves, Md. Asif Iqbal et al. [14] study created an automatic system based on image processing and machine learning that can identify and categorize potato leaf diseases. The Random Forest classifier provides a 97 % accuracy rate among them.

An image segmentation and K-means methodology-based classification strategy for potato diseases was presented in the study by Aditi Singh and Harjeet Kaur [15]. The concept of a gray level co-occurrence matrix was utilized for feature extraction, and the multi-class SVM methodology was used for classification. The suggested approach was able to achieve a 95.99 % accuracy rate on the Plant Village image dataset.

A well-liked family of machine learning techniques, Convolutional Neural Network (CNN) has been successfully used to solve a number of issues [16]. The CNN-based DL is rapidly expanding in image classification because of its hierarchical method of learning high-level from input data. DL techniques that utilized different CNN architectures were used in a number of studies to classify potato leaf diseases.

In their study, Xudong Li et al. [2] presented an integrated framework that combines the use of VGG16, ResNet50, and InceptionV3 for classification, Mask R-CNN for instance segmentation, and UNet, PSPNet, and DeepLabV3+ for semantic segmentation. Based on the experimental findings, the Mask R-CNN network achieved an Average Precision (AP) of 81.87 % in the first stage and a precision of 97.13 % in the second stage. Simultaneously, the classification model's accuracy in the second stage was 95.33 %. In the third stage, the semantic segmentation model's Mean Pixel Accuracy (MPA) was 94.24 %, and its Mean Intersection Over Union (MIoU) was 89.91 %.

In order to detect and predict potato leaf diseases, Hritwik Ghosh et al. [17] study proposed three CNN models: VGG19, DenseNet121, and ResNet50. According to the evaluation results, VGG19 was the best model for identifying diseases of potato leaves, closely followed by DenseNet121 and ResNet50.

A CNN-based model for identifying fine-grained potato diseases was presented in the study by Yu Xiaa et al. [18]. The contrastive representation features are extracted by the proposed model by appending a projection head to the Vgg16 backbone network. The experimental findings of the study demonstrated an average recognition accuracy of 97.24 %, higher than Resnet50′s 90.28 %, Resnet101′s 90.62 %, AlexNet's 93.06 %, Inception V3′s 94.44 %, and Vgg16′s 94.79 %.

One of the biggest obstacles to deep learning in image applications is the absence of labeled data. Large amounts of processing power are frequently needed for deep learning models, particularly during training. Handcrafted feature extraction can occasionally produce simpler models that require less computing power, which makes them better suited for deployment in contexts with limited resources [19]. This study's main contribution is the development of a classification model based on manually extracted features from potato leaf images. This model uses GJPs to capture crucial local features like texture, color variations, and leaf spots that are necessary for precise classification, resulting in superior classification accuracy.

## Method details

**Fig. 1:**
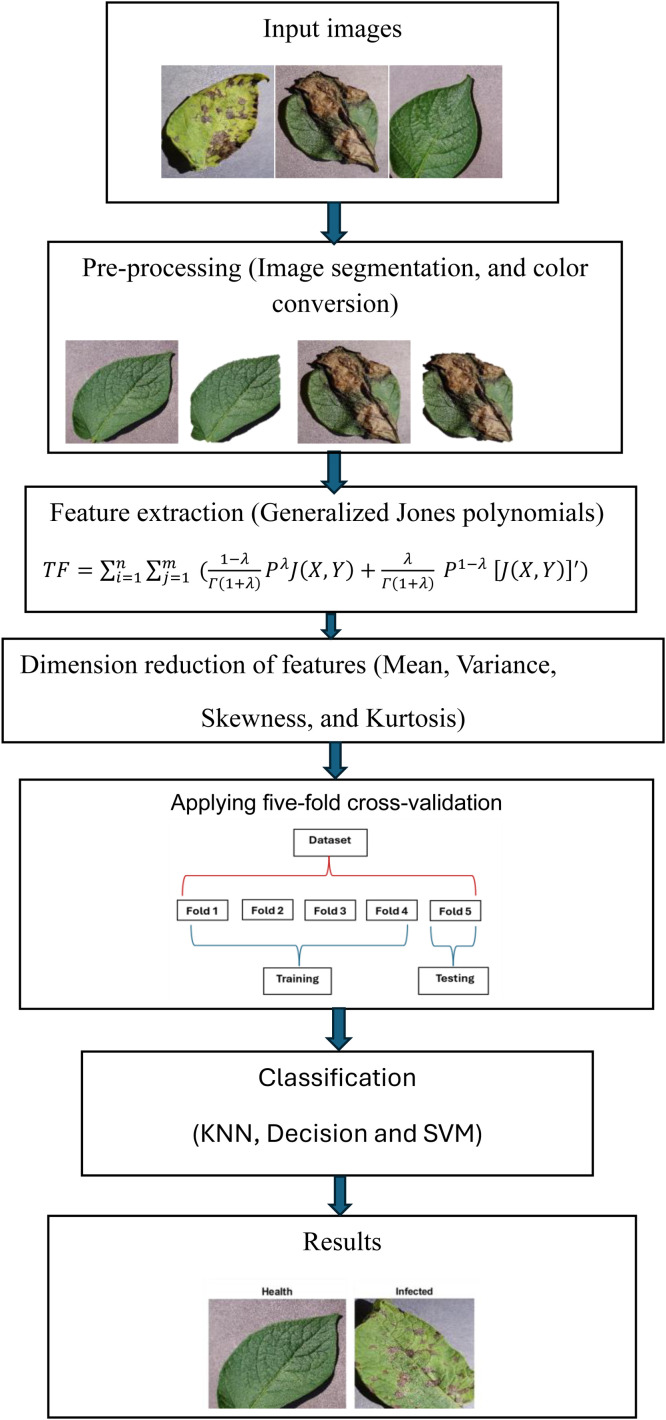
Block diagram illustrating the image preprocessing.

The classification of potato leaf diseases relies heavily on feature extraction, which makes it possible to convert leaf images into insightful and distinct representations. Texture and shape are examples of features found in traditional handcrafted techniques. These features are then fed as input into a machine learning algorithm. In order to distinguish between images of healthy and infected potato leaves, manual feature extraction techniques rely on texture analysis. In this study, features are extracted using the proposed polynomials technique. The proposed study includes dimension reduction, classification, feature extraction utilizing the proposed GJPs, data gathering, and pre-processing. Fig. 1 shows the flow mechanism for classification.

### Image acquisition

**Fig. 2:**
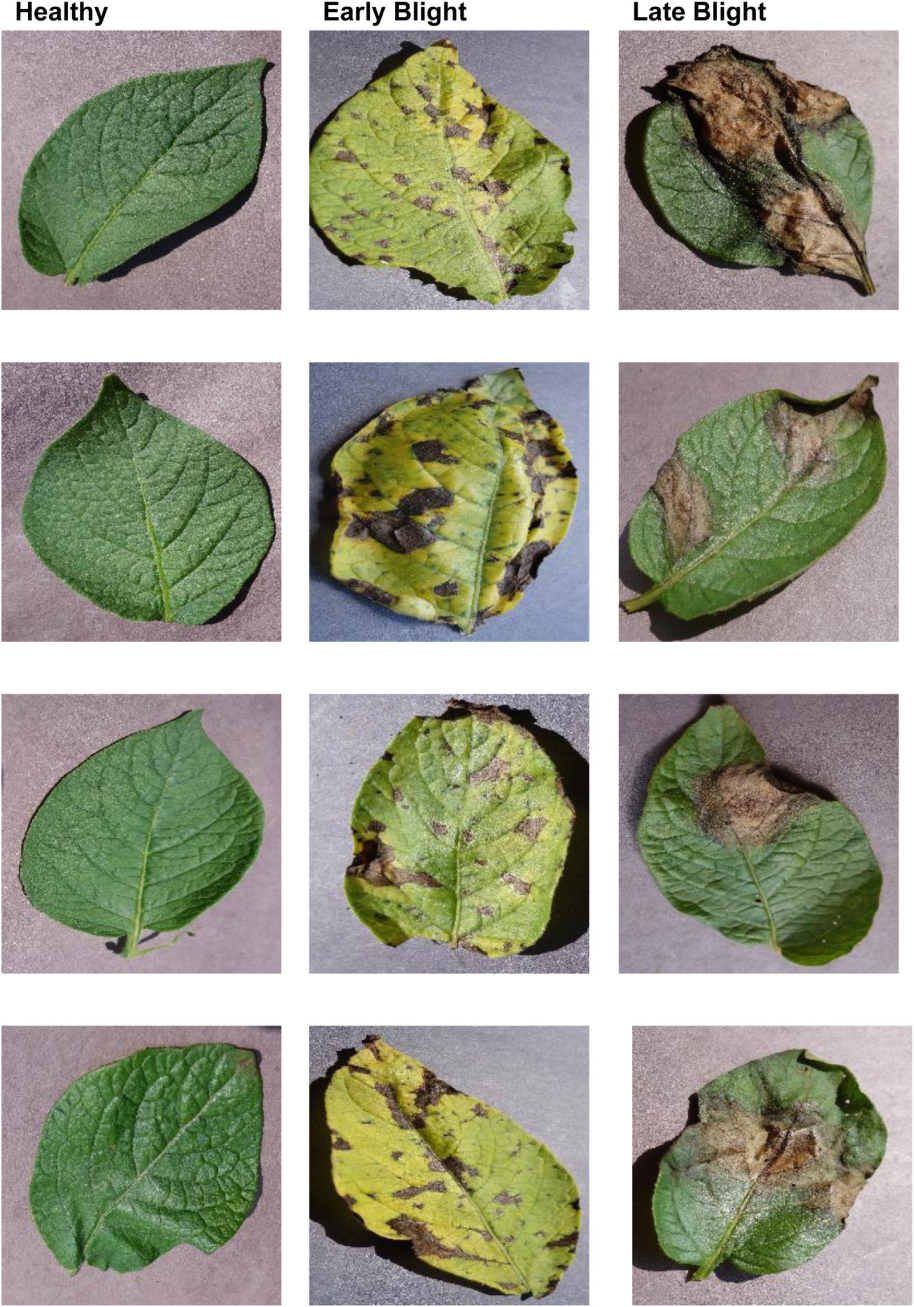
Examples of potato from Plant Village dataset [20].

The images of potato leaf infection were collected from Plant Village repository [20]. Sample of images are illustrated in Fig. 2.

### Image preprocessing

Pre-processing frequently entails converting the data into a format better suited to the classification algorithm. This study shows that by focusing on the most informative aspects of the data and reducing dimensionality, finding and choosing the most relevant features can enhance the performance of the classification model. All images of potato leaves are cropped to ensure precise and effective disease detection while maintaining consistency throughout the dataset.

### Image segmentation

**Fig. 3:**
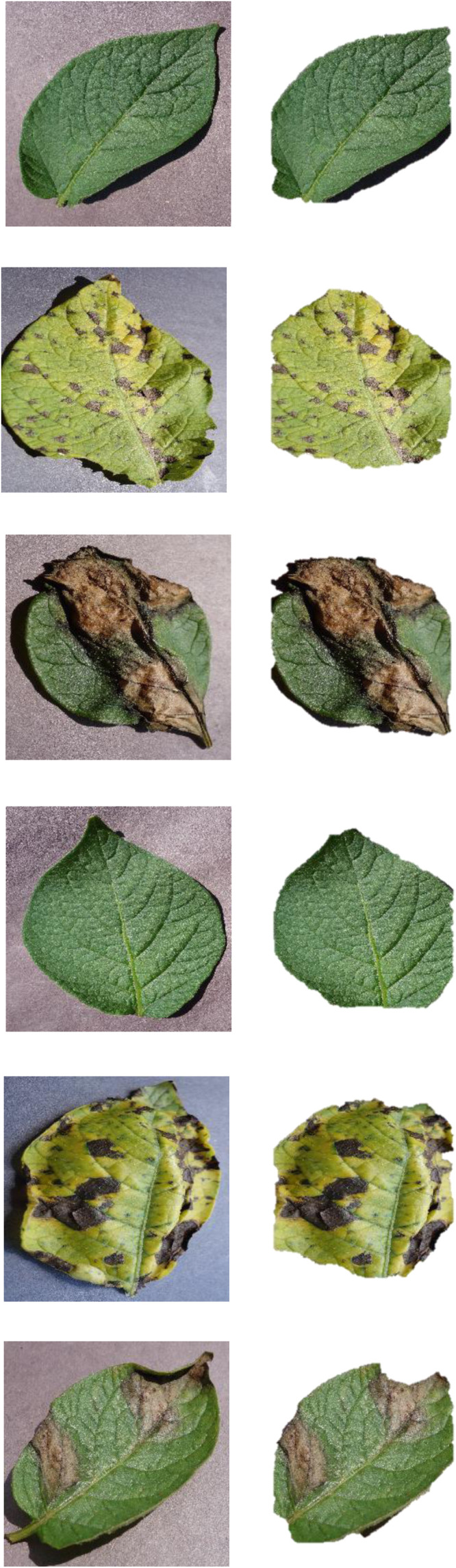
Sample of segmented images (Remove Background).

During image preprocessing, background pixels are suppressed, and the potato leaf dataset's images are segmented to extract the leaf portion from the image. This study uses a threshold-based segmentation approach wherein separate masks for green and brown are created with corresponding upper and lower threshold values. Each pixel in an image is classified as either foreground or background using threshold-based segmentation, which is based on a predetermined threshold value. To effectively separate the leaf from the background, a suitable threshold value must be chosen. The adaptive thresholding techniques used determine local threshold values by taking into account the intensities of pixels in a particular neighborhood. In situations where the illumination varies throughout the image, adaptive thresholding produced superior results. In Fig. 3, the result of the Image Segmentation step is displayed.

### Color space conversion

It is crucial to preserve color information in the potato leaf diseases because leaf diseases in potatoes frequently show up as discoloration. The original images were first converted from the RGB color space—which is composed of the red, blue, and green components—to the YCbCr color space. This YCbCr color space model representation is not dependent on the device that employs a color representation scheme based on human vision. Various factors, including the specific characteristics of the leaves, the imaging conditions, and the classification algorithm being used, influence the choice of color space components for the classification of potato leaves. Nonetheless, because of its capacity to distinguish luminance (Y) from chrominance (Cb and Cr) components, the YCbCr color space is generally a good option for leaf classification tasks [21].

### Proposed features extraction

In numerous image studies, image texture is a crucial component that greatly aids in the classification of potato leaf images. The most important feature extraction methods are polynomial-based ones that can identify even the smallest variations in an image's intensity values. The texture of potato images is recovered using a proposed GJP feature extraction model. For image feature extraction, GJPs is utilized to give the structural characteristics of images with polynomial representations. Polynomial-based image feature extraction may be able to detect even the smallest tone changes. In image processing, polynomials play a crucial role since they can distinguish between pixels and edges in an image. With a two-variable polynomial, each image can be identified. The Jones Polynomials (JPs) are an important tool in knot theory that provides basic understanding into the framework of knots and connections. Topology, algebra, and scientific theory have all benefited immensely from its discovery, and it has since established itself as a cornerstone of current mathematical research in these domains. A simple definition of JPs is that it is a Laurent polynomial in the square root of the variable.

(1) $$J\left(X,Y\right)=\;\sum_{i,j}\omega_{i,j}X^{i/2}\;Y^{j/2},$$

Where ωi,j indicates the connections (weight values) and X ^i/2^ (training targets at the position i) and Y ^j/2^ (prediction values at the position j). The following construction is shown by the GJPs: let ℓ is the fractional (arbitrary) value power such that λ ϵ [0, 1]. The operator $\Delta^{\lambda }$ is considered as a conformable differential if $\Delta^{0}$ is the self-operator and $\Delta^{1}$ is considered as a conformable differential.

Newly, Anderson and Ulness [11] offered an innovative preparation of GJPs designed by the control theory. The description has the following connotation.

Definition 1 Assume that λ ϵ [0, 1], then GJPs has in the subsequent documented.

(2) $$\;\Delta^{\lambda }g\left(x\right)=\;\nu_{1}\left(\lambda ,P\right)g\left(x\right)+\nu_{0}\;\left(\lambda ,P\right)g^{\prime }\left(x\right),$$

where $\;\Delta^{\lambda }\;$is called the fractional conformable differential operator that transforms the functiong(x) into $\;\Delta^{\lambda }g\left(x\right)$

$\nu_{1}$ and $\nu_{0}$ attain the boundaries.

(3) $$\begin{array}{ccc} & & \lim_{\lambda \;\to 0}\nu_{1}\left(\lambda ,P\right)=1,\;\lim_{\lambda \;\to 1}\nu_{1}\left(\lambda ,P\right)=0,\\ & & \lim_{\lambda \to 0}\nu_{0}\left(\lambda ,P\right)=0,\;\lim_{\lambda \;\to 1}\nu_{0}\left(\lambda ,P\right)=1.\\ & & \nu_{1}\left(\lambda ,P\right)=\frac{1-\lambda }{\Gamma \left(1+\lambda \right)}\;P^{\lambda },\;\nu_{0}\;\left(\lambda ,P\right)=\frac{\lambda }{\Gamma \left(1+\lambda \right)}\;P^{1-\lambda } \end{array}$$

By substituting (1), (3) into Eq. (2) the fractional conformable can be obtained

(4) $$\begin{array}{ccc} & & \Delta^{\lambda }\;J\left(X,Y\right)=\;\nu_{1}\left(\lambda ,P\right)\;J\left(X,Y\right)+\nu_{0}\;\left(\lambda ,P\right)\;{\left[J\left(X,Y\right)\right]}^{\prime }\\ & & \Delta^{\lambda }\;J\left(X,Y\right)=\;\frac{1-\lambda }{\Gamma \left(1+\lambda \right)}P^{\lambda }J\left(X,Y\right)+\frac{\lambda }{\Gamma \left(1+\lambda \right)}\;P^{1-\lambda }\;{\left[J\left(X,Y\right)\right]}^{\prime } \end{array}$$

For one image block the texture feature is given by

(5) $$TF=\sum_{i=1}^{n}\sum_{j=1}^{m}\;\left(\frac{1-\lambda }{\Gamma \left(1+\lambda \right)}P^{\lambda }J\left(X,Y\right)+\frac{\lambda }{\Gamma \left(1+\lambda \right)}\;P^{1-\lambda }\;{\left[J\left(X,Y\right)\right]}^{\prime }\right)\;$$

where${\left[J(X,Y)\right]}^{{}^{\prime }}\;$indicates the image derivative, *J(X,Y)* is the input image, P denotes pixel probability in the two directions, and λ is a scaling factor that controls the influence of the derivative which represents the fractional conformable number experimentally fixed to 0.5.

By adding the derivative of an image to the original image can enhance certain features, particularly edges, and can affect the overall appearance of the image in several ways, depending on how the derivative is computed. The effect of Adding Derivative to Image are the edge Enhancement, Since the gradient highlights areas of rapid intensity change (edges), adding it to the original image tends to enhance the edges. Moreover, the overall contrast of the image can increase, making features more prominent. The input image *J(X,Y)* is first divided into non-overlapping blocks for the feature extraction.

Fig. 4

**Fig. 4:**
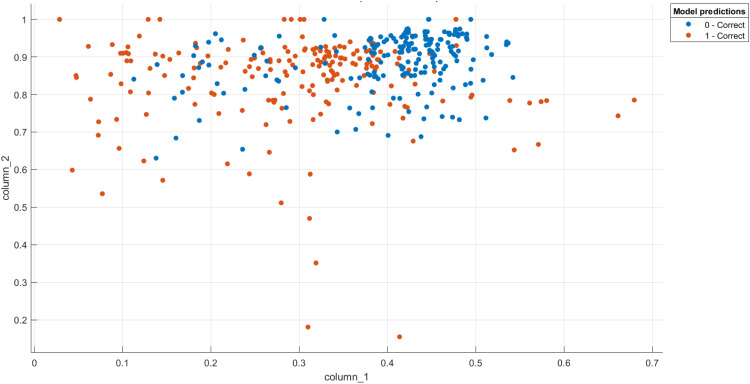
The distribution of the two classes features (potato leaves as healthy (blue dots) or infected (red dots)).

The dimensionality reduction of features is used to lower computational costs and resource allocation once the suggested texture features expression is derived from each image using Eq (5). This ensures that the algorithm is running at its most effective and ideal parameters. The ``Mean'', ``Variance'', ``Skewness'', and ``Kurtosis'' measurements are used in the current work's suggested feature reduction approach to lower the dimensionality of the retrieved data in each image.

For features F of M scalar observations, the “Mean” is defined as

(6) $$Mn=\frac{1}{M}\sum_{I=1}^{M}F_{i}$$

The definition of a ``Variance'' is

(7) $$Vc=\frac{1}{M-1}\sum_{i=1}^{NM}{\left|F_{i}-\mu \right|}^{2}$$

where µ is the “Mean” of F_i_

A measure known as ``skewness'', which is defined as the asymmetry of the feature around the feature mean,

(8) $$Ss=\frac{V{\left(x-\mu \right)}^{3}}{St^{3}}$$

where St is the ``Standard deviation.''

(9) $$Ks=\frac{V{\left(x-\mu \right)}^{4}}{St^{4}}$$

The ‘Standard deviation’ is described as

(10) $$St=\sqrt{\frac{1}{M-1}}\sum_{i=1}^{M}{\left|F_{i}-\mu \right|}^{2}$$

### Classification

The accuracy, precision, recall, and F1 classification of potato leaf infections are compared in this study for different classification algorithms, including K-Nearest Neighbors (KNN), Decision Tree, and Support Vector Machine (SVM) classifiers. The KNN algorithm is a simple classifier of machine learning techniques that works very well for basic recognition problems. When evaluating a new testing sample, KNN determines its class by analyzing the distances between the sample and its K nearest neighbors in the training dataset. The decision trees are valued for their simplicity and ease of interpretation, making them a potent and intuitive tool for both regression and classification tasks. Decision trees have an easy-to-understand, visualizable tree structure that makes it easier to understand the model and explain it to others. In addition, the decision trees don't require a lot of data preprocessing to function with categorical or numerical data.

### Evaluation metrics

The following criteria were used to assess the classification results:

(11) $$\text{Accuracy}\;=\frac{\text{TP}\;+\;\text{TN}}{\text{TP}\;+\;\text{FP}\;+\;\text{TN}\;+\;\text{FN}}$$

(12) $$\text{Precision}\;=\frac{\text{TP}}{\text{TP}\;+\;\text{FP}}\;$$

(13) $$\text{Recall}\;=\frac{\text{TP}}{\text{TP}\;+\;\text{FN}}$$

(14) $$F1\;=\frac{2\text{PR}}{P\;+\;R}$$

where True Positive (TP) refers to successfully expected positive values, False Positive (FP) to mistakenly shown positive values, True Negative (TN) to correctly predicted negative values, and False Negative (FN) to incorrectly predicted negative values.

## Method validation

**Fig. 5:**
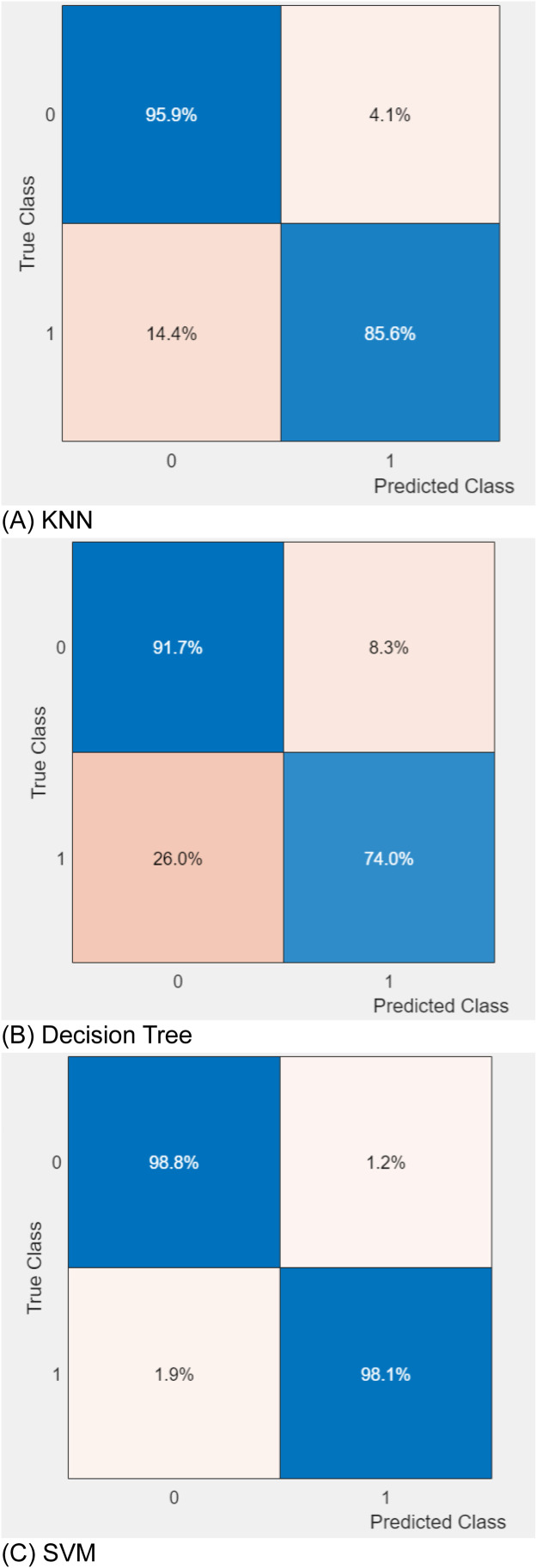
Confusion matrixes for testing results.

**Fig. 6:**
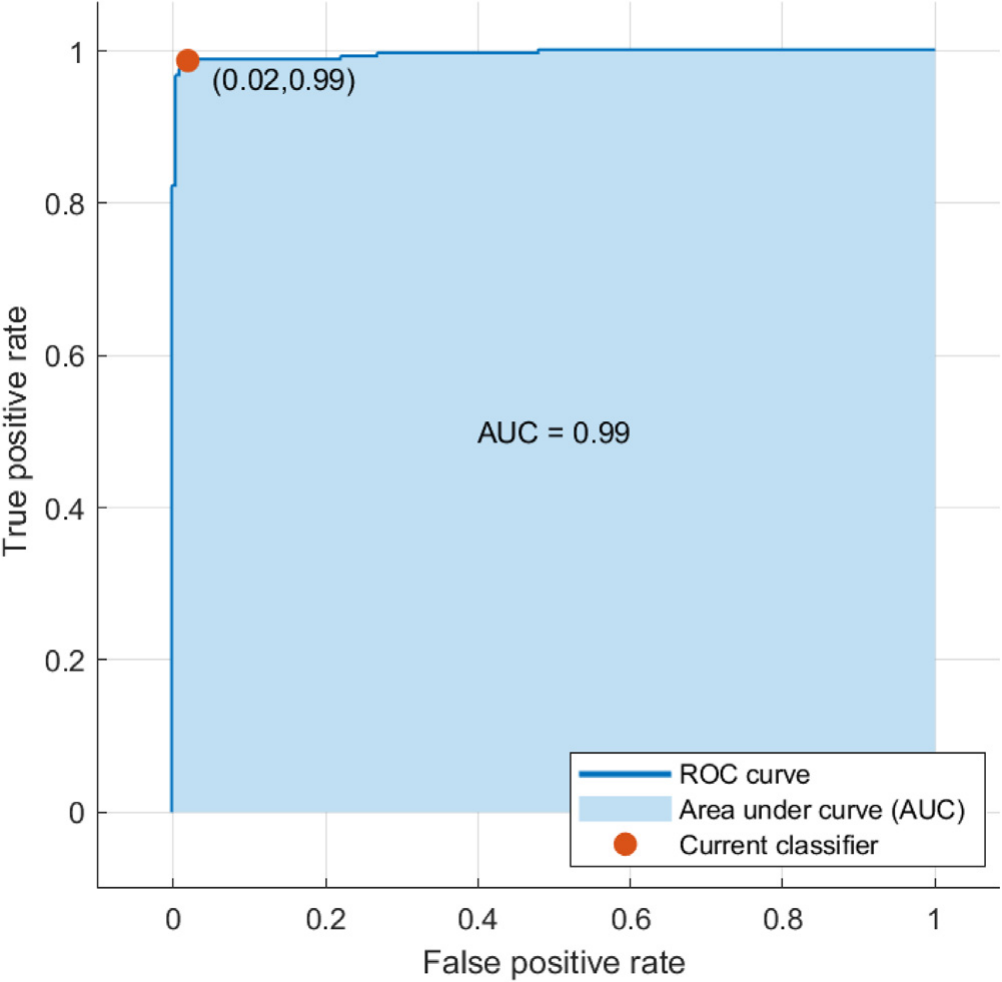
The area under the curves (AUCs).

To conduct each test, MATLAB 2021b was employed. 5-fold cross-validation is the technique used in this study. With 30 % of the images used for testing and 70 % for training during each iteration, the dataset is split into five subsets and the main process is run five times. In order to determine which classifier performs best in terms of classification, three different classifiers are trained using the features retrieved by the recommended GJPs method in machine learning for the classification of potato infections. The confusion matrices for the algorithms are displayed in Fig. 5, where the KNN algorithm and SVM classifiers were found to be the most effective. This indicates a direct correlation between the quality of the extracted features and the final classification outcome in the classification task. Table 1 presents the comprehensive findings. The tested machine learning techniques had accuracy levels of 92.40 % (KNN), 89.70 (Decision Tree) and 98.45 % (SVM). Of the three machine learning methods examined, the KNN and SVM classifiers yielded the best classification results. The ``Area Under the Curve'' (AUC) and the ``receiver operating Characteristic Curve'' (ROC) are two more methods for validating classification results. Fig. 6 displays the ROC curve for the suggested model. It is possible to conclude that the categorization classes are more effectively divided because the AUC is 0.99 (higher is better).

**Table 1:** The classification results by the KNN, Decision tree and SVM, classifiers.

Classifiers	Accuracy %	Precision %	Recall %	F1 %	Hyperparameters
KNN	90.75	86.94	95.90	91.203	Number of neighbors: 10 Distance metric: Euclidean Distance weight: Equal
Decision Tree	82.85	77.90	91.70	84.24	Maximum number of splits:100 Split criterion: Gini’s index
SVM	98.45	98.11	98.80	98.45	Kernal function: Cubic kernel Kernal scale (γ): Auto Box constraint level: 1 Multiclass method: One-vs-One

A comparison with earlier research that looked into the categorization of potato leaves to enhance the distinction between healthy and infected cases is provided to demonstrate the effectiveness of the suggested feature extraction technique. The performance comparison with another study using the same image dataset (the PlantVillage dataset) is displayed in Table 2. The following techniques have been developed for the classification of potato leaf diseases.

**Table 2:** Comparison of performance using the PlantVillage potato dataset.

Method	Classifiers	Testing Accuracy %
Islam M et al. [13]	SVM	95
Iqbal MA et al. [14]	Random Forest classifier	97
Singh A,and Kaur H [15]	Multi-class SVM	95.99
Xudong Li et al. [2]	VGG16, ResNet50, and InceptionV3	95.33
Ghosh H et al. [17]	VGG19	98.77
Xia Y et al. [18]	VGG16	97.24
Proposed method	SVM	98.45

Islam M et al. [13] presented an approach that combines image processing and machine learning. The GLCM was used to extract statistical texture features like contrast, correlation, energy, and homogeneity. With a 95 % accuracy rate, the SVM classifier can classify diseases across 300 images.

Iqbal MA et al. [14] study created an automatic system based on image processing and machine learning that can identify and categorize potato leaf diseases. The Random Forest classifier provides a 97 % accuracy rate among them.

Singh A and Kaur H [15]. proposed an image segmentation and K-means methodology-based classification strategy for potato diseases. The multi-class SVM methodology was used for classification, while the gray level co-occurrence matrix concept was applied for feature extraction. The suggested approach was able to achieve a 95.99 % accuracy rate on the Plant Village image dataset.

Xudong Li et al. [2] presented an integrated framework in their work that combines the application of Mask R-CNN for instance segmentation, VGG16, ResNet50, and InceptionV3 for classification, and UNet, PSPNet, and DeepLabV3+ for semantic segmentation. The classification model's accuracy was 95.33

Ghosh H et al. [17] study proposed three CNN models: VGG19, DenseNet121, and ResNet50. The evaluation results show that for identifying potato leaf diseases, VGG19 emerged as the top performer, closely followed by DenseNet121 and ResNet50. A CNN-based model for identifying fine-grained potato diseases was presented in the study by Yu Xiaa et al. [18]. The contrastive representation features are extracted by the proposed model by appending a projection head to the Vgg16 backbone network. The study's experimental results showed an average recognition accuracy of 97.24 %. When compared to alternative approaches, the suggested GJPs feature extraction model increased classification accuracy, as shown in Table 2.

The results in Table 2 showed how successful the suggested feature extraction method is, showing that it produced the best results for detecting potato leaf disease. The study by Hritwik Ghosh et al. [17] used a deep neural network model to classify potato plant leaf diseases, which improved classification accuracy but required more processing power. The findings show that the suggested method achieves a respectable classification accuracy with the least number of feature dimensions. This shows that as a texture descriptor for image classification, the suggested feature extraction method is practical and trustworthy. By using computer vision techniques to detect diseases early and stop them from spreading across fields, crop yields are improved. Appropriate disease identification and classification are essential for averting unforeseen circumstances. In order to extract the most important features from potato leaves for the classification of potato infections using three machine learning algorithms—KNN, Decision Tree algorithms, and SVM—a new feature extraction method based on GJPs was proposed in this study. Comparative tests revealed that the SVM produced the best classification outcomes out of all the classifiers. Plant disease images can also be identified and categorized using the approach presented in this study. With the suggested feature extraction method, high classification accuracy has been achieved while the complexity of the training models has reduced. Furthermore, the results showed that, when compared to previous studies on potato leaf diseases, the experimental performance of the proposed feature extraction was superior. Future research will examine multiple infection types, which will be more useful in real-world scenarios. The suggested approach to classifying potato infections has a drawback in that it is susceptible to artifacts in images of potato leaves, which could lead to incorrect classification because some diseases exhibit similar symptoms on the leaves.

## Limitations

The Jones polynomial transformation was applied after a number of important preprocessing steps. Errors in these procedures may result in inaccurate polynomial features that are extracted, which would ultimately lower classification performance. Some of these limitations may be decreased by integrating Jones polynomial features with other reliable techniques or by using them as supplemental features in conjunction with machine learning models.

## Ethics statements

This article does not contain any studies with human or animal participants.

## Funding

This research received no external funding.

## Declaration of competing interest

The authors declare that they have no known competing financial interests or personal relationships that could have appeared to influence the work reported in this paper.

## Data Availability

Data will be made available on request.

## References

[1] Ala'a R., Ibrahim R.W (2024). Classification of tomato leaf images for detection of plant disease using conformable polynomials image features. MethodsX.

[2] Li X., Zhou Y., Liu J., Wang L., Zhang J., Fan X. (2022). The detection method of potato foliage diseases in complex background based on instance segmentation and semantic segmentation. Front Plant Sci.

[3] Finance Y. Saudi Arabia agriculture Market. 2024.

[4] Math R.M., Dharwadkar N.V. (2022). Early detection and identification of grape diseases using convolutional neural networks. J. Plant Dis. Prot..

[5] Vishnoi V.K., Kumar K., Kumar B. (2022). A comprehensive study of feature extraction techniques for plant leaf disease detection. Multimed Tools Appl.

[6] Alzahem A., Boulila W., Koubaa A., Khan Z., Alturki I. (2023). Improving satellite image classification accuracy using GAN-based data augmentation and vision transformers. Earth Sci Inf..

[7] Jothi G., Inbarani H.H., Azar A.T., Koubaa A., Kamal N.A., Fouad K.M. (2020). Improved dominance soft set based decision rules with pruning for Leukemia image classification. Electron. (Basel).

[8] Almasoud A.S., Abdelmaboud A., Alsubaei F.S., Hamza M.A., Yaseen I., Abaker M. (2022). Deep learning with image classification based Secure CPS for healthcare sector. Comput. Mater. Contin..

[9] Saba T., Bokhari S.T.F., Sharif M., Yasmin M., Raza M. (2018). Fundus image classification methods for the detection of glaucoma: a review. Microsc, Res, Tech.

[10] Xiong Y., Liang L., Wang L., She J., Wu M. (2020). Identification of cash crop diseases using automatic image segmentation algorithm and deep learning with expanded dataset. Comput. Electron. Agric..

[11] Anderson D., Camrud E., Ulness D. (2019). On the nature of the conformable derivative and its applications to physics. J Fract Calc Appl.

[12] Tan L., Lu J., Jiang H. (2021). Tomato leaf diseases classification based on leaf images: a comparison between classical machine learning and deep learning methods. AgriEngineering.

[13] Islam M., Dinh A., Wahid K., Bhowmik P. (2017). 2017 IEEE 30th canadian conference on electrical and computer engineering (CCECE).

[14] Iqbal M.A., Talukder K.H. (2020). 2020 international conference on wireless communications signal processing and networking (WiSPNET).

[15] Singh A., Kaur H. (2021). IOP Conference Series: Materials Science and Engineering.

[16] Aldawish I., Jalab Hamid A (2024). Deep and hand-crafted features based on Weierstrass elliptic function for MRI brain tumer classification. J. Intellienent Syst. DE GRUYTER.

[17] Ghosh H., Rahat I.S., Shaik K., Khasim S., Yesubabu M. (2023). Potato leaf disease recognition and prediction using convolutional Neural networks. EAI Endorsed Trans. Scalable Inf. Syst..

[18] Xia Y., Tang M., Tang W. (2023). Fine-grained potato disease identification based on contrastive convolutional neural networks. Appl. Artif. Intell..

[19] Ibrahim R.W., Hasan A.M., Jalab H.A. (2018). A new deformable model based on fractional Wright energy function for tumor segmentation of volumetric brain MRI scans. Comput Methods Programs Biomed.

[20] PlantVillage. 2019.

[21] Jalab H.A., Subramaniam T., Ibrahim R.W., Kahtan H., Noor N.F.M. (2019). New texture descriptor based on modified fractional entropy for digital image splicing forgery detection. Entropy.

